# Microbiomics and volatile metabolomics-based investigation of changes in quality and flavor of oat (*Avena sativa* L.) silage at different stages

**DOI:** 10.3389/fpls.2023.1278715

**Published:** 2023-11-06

**Authors:** Xiaochen Deng, Yushan Jia, Gentu Ge, Zhijun Wang, Mingjian Liu, Jian Bao, Muqier Zhao, Qiang Si, Yichao Liu, Weixuan Zhao

**Affiliations:** ^1^ Key Laboratory of Forage Cultivation, Processing and High Efficient Utilization of Ministry of Agriculture and Rural Affairs, Inner Mongolia Agricultural University, Hohhot, China; ^2^ Key Laboratory of Grassland Resources, Ministry of Education, Inner Mongolia Agricultural University, Hohhot, China

**Keywords:** oats, microbial community, volatile metabolites, flavor, fermentation quality

## Abstract

**Objective:**

This study aimed to analyze the fermentation quality, microbial community, and volatile metabolites of oat silage harvested at two different stages, while examining the correlation between microorganisms and volatile metabolites.

**Methods:**

Oats were harvested at two growth stages (pre-heading [PRH] and post-heading [POH] stages), followed by 90 days of natural fermentation, with 6 replicates per treatment. Pre- and post-silage samples were randomly selected for nutrient composition, fermentation parameters, microbial population, and high-throughput sequencing analysis. Volatile metabolomics analysis was also performed on samples after 90 days of fermentation to detect differences in flavor quality after silage.

**Results:**

The effect of growth stage on the nutrient content of oats was significant, with pre-heading oats having higher crude protein and post-heading oats having higher water soluble carbohydrates content (*p* < 0.05). Following a 90-day fermentation period, the pH and ammonia nitrogen/total nitrogen levels in the PRH-90 (silage from pre-heading oats after 90 days of fermentation) group demonstrated a significant decrease (*p* < 0.05), whereas the lactic acid content was notably higher compared to the POH-90 (silage from post-heading oats after 90 days of fermentation) group (*p* <0.05). *Lactiplantibacillus* dominated in the PRH-90 group and *Enterococcus* dominated in the POH-90 group, with abundances of (> 86%) and (> 87%), respectively. The differential volatile metabolites of the two treatment groups were dominated by esters and terpenoids, and the differences in flavor were mainly concentrated in sweet, green, and fruity odors. The results of Kyoto encyclopedia of genes and genomes pathway enrichment analysis demonstrated three major metabolic pathways: phenylpropanoid biosynthesis, phenylalanine metabolism, and biosynthesis of secondary metabolites. Specific microorganisms were significantly correlated with flavor indicators and flavor metabolites. *Lactiplantibacillus* was significantly positively correlated with flavor substances indicating sweet and fruity flavors, contributing to good flavor, while *Enterococcus* was significantly and positively correlated with flavor substances indicating bad flavors.

**Conclusion:**

In summary, growth stage had significant effects on nutritional components, fermentation parameters and flavor quality of oats, with the fermentation process dominated by *Lactiplantibacillus* leading to good flavor, while the fermentation process dominated by *Enterococcus* led to the development of poor flavor.

## 1 Introduction

As an important grain-feed crop for livestock, oats (*Avena sativa* L.) are widely cultivated worldwide and have the advantages of high nutritional value, high grass yield, and good palatability (Zhou et al., 1999). Ensiling oats, a common preservation method, can be preserve oats for a long period of time, and also improves palatability and the organoleptic quality of oats (Nilsson and Rydin, 1963).

Ensiling refers to a technique of reducing the pH value of raw materials through microbial fermentation, inhibiting the growth and reproduction of detrimental microorganisms, in order to maximize the preservation of its nutrients and extend the shelf life of the raw material (Mcdonald et al., 1991). As a crop rich in protein, fiber, and trace elements, oats can be better preserved and utilized for its nutrient content after ensiling. However, microbial activity and metabolites during ensiling may have an impact on the quality and flavor of oats (Limin et al., 2018).

In recent years, microbiomics and volatile metabolomics have emerged as important branches of modern biotechnology, finding extensive applications in the fields of food science and nutrition (Shi et al., 2022; Zhao et al., 2022). Microbiomics provides insight into the species, abundance and functions of different microorganisms through the study of microbial communities, which in turn reveals their association with the quality and flavor of fermented products (Paraskevi et al., 2020). Volatile metabolomics, on the other hand, can explore the flavor characteristics of fermented products and their relationship with microbial metabolic activities by analyzing the composition and variation of volatile compounds in food (Iijima, 2014). The investigation of microbial communities and volatile metabolites during oat ensiling is essential to understand the complex biochemical processes involved in the ensiling process. Microorganisms, particularly lactic acid bacteria, assume a crucial role in the fermentation process, producing organic acids and other metabolites that contribute to the preservation and flavor development of silage oats (Liu M. et al., 2022). In addition, the composition and abundance of volatile compounds may vary depending on silage conditions, oat fertility and microbial interactions, ultimately affecting the sensory characteristics of the final product. Previous studies have focused on oat fertility, moisture content, and exogenous additives (Gardner and Wiggans, 1961; Mustafa and Seguin, 2003; Jian et al., 2015; Gomes et al., 2019; Xu et al., 2022), with fewer studies on oat silage odor after oat silage fermentation, and no in-depth studies on the relationship between microbial activity and flavor development during the fermentation process.

The objective of this study is to investigate the alterations in quality and flavor of oats following the silage process at different stages using microbiomics and volatile metabolomics. Through the analysis of microbial communities and volatile metabolites, we aim to identify the patterns and dynamics of microorganisms and volatile compounds during oat ensiling, which can lay a scientific foundation for the improvement of oat ensiling techniques and enhancing silage quality.

## 2 Methods and materials

### 2.1 Silage preparation

The oats were harvested on August 22, 2022 (pre-heading stage) and September 6, 2022 (post-heading stage), respectively, in Ar Horqin Banner, Chifeng City, Inner Mongolia Autonomous Region, China (43°21′43″-45°24′20″N, 119°02′15″-121°01′E). A part of the harvested oat material was directly returned to the laboratory for the determination of fresh oat material, while the other part was naturally air-dried to the substance with rough 70% moisture content, and then the sample was cut into 2-3cm in length. The experiment was divided into four groups with six bags (250×360mm polythene plastic bag) per group, each bag containing 400 g without any additives. The bags vacuum sealed for storage (Type: DZ-500/2E; Hefei Hanjie Packaging Machinery Inkjet Co., Ltd., Hefei, China) and opened and sampled after 90 d of storage at room temperature for the subsequent analysis. The different treatment groups are named as follows: PRH-fm, fresh oat material at pre-heading stage; POH-fm, fresh oat material at post-heading stage; PRH-90, silage from pre-heading oats after 90 days of fermentation; POH-90, silage from post-heading oats after 90 days of fermentation.

### 2.2 Laboratory analysis

Samples of 10 g each were collected from each treatment group at 0 days and 90 days. Subsequently, 90 mL of distilled water was added, and the mixture was homogenized for two minutes by a homogenizer (Model: HX-4, Shanghai Huxi Industrial Co., Ltd., China). After filtration of the resulting extract, the pH value was measured using an acidity meter (Model: S400-B, Mettler-Toledo, LLC, America). The remaining materials were placed in an oven at 115°C for 15 minutes and then dried at 65°C for 48 hours for weighing dry matter (DM) content. The dried materials were ground into powder and stored separately. The Kjeldahl method was employed to analyze crude protein (CP) content (Sun et al., 2021). An ANKOM fiber analyzer was utilized to quantify neutral detergent fiber (NDF) and the acid detergent fiber (ADF) (Model: A2000i; Beijing Anke Borui Technology Co., Ltd., China), and the measurement of soluble carbohydrates (WSC) was performed through anthrone-sulfuric acid colorimetry (Murphy, 1958). Determination of lactic acid (LA) and acetic acid (AA) in silage after 90 days of fermentation was accomplished by high performance liquid chromatography (Fu et al., 2022). Determination of ammonia nitrogen (NH_3_-N) concentration used the phenol hypochlorite method according to Broderick and Kang (1980).

### 2.3 Enumeration of microbial community

Ten g of fresh and silage oat samples were collected, and 90 mL of sterile water was added. The mixture was homogenized for 2 minutes using a homogenizer (Model: HX-4, Shanghai Huxi Industrial Co., Ltd., China), and the resulting bacterial solution was obtained after filtration. Culture media (Guangzhou Huankai Microbial Science and Technology Co., Ltd., Guangzhou, China) were used to isolate and enumerate various microorganisms. The culture medium for lactic acid bacteria was De Man Rogosa Sharpe agar culture medium, while nutrient agar culture medium was used for aerobic bacteria, iron-methylene blue agar culture medium for coliform bacteria, and potato glucose agar culture medium for mold and yeast. The quantification of microbial communities was performed using the plate counting method. The number of colonies was the number of viable microorganisms in the colony forming unit (cfu)/g fresh substance (FM). The number of viable microorganisms in colony forming unit (cfu)/g of fresh matter (FM) was determined by counting the colonies.

### 2.4 DNA extraction and PCR amplification and sequencing

Total microbial genomic DNA was extracted from homogenized experimental fresh oat samples and silage oat samples using the E.Z.N.A.^®^ soil DNA Kit (Omega Bio-tek, Norcross, GA, U.S.) according to the manufacturer’s instructions. The quality and concentration of DNA were determined by 1.0% agarose gel electrophoresis and a NanoDrop2000 spectrophotometer (Thermo Scientific, United States). The hypervariable region V3-V4 of the bacterial 16S rRNA gene was amplified with primer pairs 799F and 1193R (Liu et al., 2016) by T100 Thermal Cycler PCR thermocycler (BIO-RAD, USA). The PCR reaction mixture consisted of 4 μL of 5× Fast Pfu buffer, 2 μL of 2.5 mM dNTPs, 0.8 μL of each primer (5 μM), 0.4 μL of Fast Pfu polymerase, 0.2 μL of BSA, 10 ng of template DNA, and ddH2O, resulting in a final volume of 20 µL. Following the manufacturer’s instructions, the PCR product was extracted and purified from a 2% agarose gel using the PCR Clean-Up Kit (YuHua, Shanghai, China). Subsequently, the purified product was quantified using the Qubit 4.0 system, as per the manufacturer’s protocol (Thermo Fisher Scientific, USA). Sequencing data for the 16S rRNA gene sequence of the oat samples in the two treatment groups were uploaded and stored in NCBI BioProject, and the accession number can be found under PRJNA1005624.

### 2.5 Analysis of volatile metabolites present in oat silage samples

#### 2.5.1 Sample preparation and treatment

The oat materials were harvested, weighed, and promptly frozen in liquid nitrogen for preservation. Subsequently, they were stored at -80°C until required for analysis. To prepare the samples, the frozen oat materials were ground into a fine powder using liquid nitrogen.

For each analysis, 500 mg (1 mL) of the powdered oat sample was immediately transferred into a 20 mL headspace vial (Agilent, Palo Alto, CA, USA). The vial was supplemented with 10μL of saturated NaCl solution (50μg/mL) to prevent enzyme reactions. The vials were tightly sealed using crimp-top caps equipped with TFE-silicone headspace septa (Agilent). Prior to solid-phase microextraction (SPME) analysis, each vial was placed in an oven set at 60°C for 5 minutes. Subsequently, a 120 µm DVB/CWR/PDMS fiber (Agilent) was exposed to the headspace of the sample for 15 minutes at 60°C.

#### 2.5.2 GC-MS conditions

The identification and quantification of volatile organic compounds (VOCs) were carried out using an Agilent Model 8890 gas chromatograph coupled with a 7000D mass spectrometer (Agilent). The GC system was equipped with a DB-5MS (5% phenyl-polydimethylsiloxane) capillary column measuring 30 m × 0.25 mm × 0.25 μm. Helium gas was utilized as the carrier gas, flowing at a linear velocity of 1.2 mL/min. The injector temperature was maintained at 250°C, while the detector temperature was set at 280°C. To achieve separation and analysis, the oven temperature was programmed as follows: initial temperature of 40°C for 3.5 minutes, followed by a ramp of 10°C/min to 100°C, then a ramp of 7°C/min to 180°C, and finally a ramp of 25°C/min to 280°C. The temperature was held at 280°C for 5 minutes.

### 2.6 Statistical analysis

Significant differences in the test materials were analyzed using SAS 9.2, and the 0.05 level was considered to be the least significance level between the treatment groups. Unsupervised principal component analysis (PCA) was conducted using the prcomp statistical function in R (www.r-project.org). The hierarchical cluster analysis (HCA) results for samples and metabolites were generated using the Complex Heatmap R package. Microbiota and metabolome data were performed using an online platform of Majorbio Cloud Platform (https://cloud.majorbio.com/page/tools/).

## 3 Results

### 3.1 Characteristics of fresh and silage oats at different stages

The nutritional components, fermentation products, and microbial populations of whole oats before and after silage at different stages are shown in **Table 1**. Distinct variations were observed in the nutritional components, fermentation products, and microbial population of oats between the pre-heading and post-heading stages. The NDF (628.27 ± 8.56), ADF (381.98 ± 17.10), and WSC (50.63 ± 1.40) of fresh oats at the post-heading stage significantly increased compared to the pre-heading stage, while CP (10.38 ± 0.41) significantly decreased (*p* < 0.05). Following a 90-day fermentation period, there was a significant decrease in the content of the CP, NDF, ADF, and WSC in silage oats harvested at both pre-heading and post-heading stages (*p* < 0.05). Silage oats at pre-heading stage had lower NDF (464.71 ± 11.83), ADF (296.53 ± 0.55), WSC (17.54 ± 0.51), but higher CP (10.80 ± 0.31) than at post-heading stage (*p* < 0.05). Comparing the fermentation products of the two stages, pH, AA, and NH3-N were significantly lower in pre-heading oats after ensiling compared to post-heading oats, and LA and LA/AA were found to be significantly higher in pre-heading oats compared to post-heading oats (*p* < 0.05). The results of microbial plate count analysis revealed no statistically significant differences in the populations of aerobic bacteria and coliform bacteria attached to fresh oats at the two stages (*p* < 0.05), while lactic acid bacteria and yeast attached to fresh oats at the pre-heading stage were lower than those attached to fresh oats at post-heading stage (*p* < 0.05). After ensiling, the populations of lactic acid bacteria and aerobic bacteria increased significantly in oats at the pre-heading stage, while the opposite was observed in oats at the post-heading stage (*p* < 0.05). No presence of molds was detected in any of the treatment groups.

**Table 1:** Nutritional components, fermentation products, and microbial populations of whole oats harvested at different stages before and after ensiling.

Parameters analyzed	PRH		POH	
	PRH-fm	PRH-90	POH-fm	POH-90
Nutritional Components				
DM, g/kg	136.40 ± 15.47d	284.43 ± 6.89b	205.72 ± 4.69c	332.93 ± 2.95a
CP, g/kg DM	12.91 ± 0.45a	10.80 ± 0.31b	10.38 ± 0.41b	8.74 ± 0.44c
NDF, g/kg DM	501.54 ± 11.89c	464.71 ± 11.83d	628.27 ± 8.56a	553.45 ± 4.95b
ADF, g/kg DM	308.04 ± 22.58c	296.53 ± 0.55c	381.98 ± 17.10a	340.74 ± 6.08b
WSC, g/kg DM	30.32 ± 1.32c	17.54 ± 0.51d	50.63 ± 1.40a	35.33 ± 1.14b
Fermentation Products				
pH	6.26 ± 0.06a	4.73 ± 0.06d	5.95 ± 0.20b	5.57 ± 0.11c
LA, g/kg DM	/	53.66 ± 10.74a	/	30.43 ± 4.39b
AA, g/kg DM	/	8.86 ± 1.74b	/	14.41 ± 0.83a
LA/AA	/	6.34 ± 2.29a	/	2.13 ± 0.42b
AN/TN	/	6.22 ± 0.45b	/	9.06 ± 0.35a
Microbial population				
Lactic acid bacteria (log_10_cfu/g FM)	3.86 ± 0.48c	5.90 ± 0.29a	5.10 ± 0.26b	4.66 ± 0.55b
Aerobic bacteria (log_10_cfu/g FM)	5.89 ± 0.21a	6.20 ± 0.86a	6.63 ± 0.40a	4.77 ± 0.31b
Coliform bacteria (log_10_cfu/g FM)	4.77 ± 0.68a	ND	5.28 ± 0.80a	ND
Yeast (log_10_cfu/g FM)	2.37 ± 0.32d	6.10 ± 0.09a	4.11 ± 0.07c	4.71 ± 0.24b
Molds (log_10_cfu/g FM)	ND	ND	ND	ND

DM, dry matter; CP, crude protein; NDF, neutral detergent fiber; ADF, acid detergent fiber; WSC, water soluble carbohydrates; pH, potential of hydrogen; LA, lactic acid; AA, acetic acid; AN, ammonia nitrogen; TN, total nitrogen; cfu, colony forming unit; FM, fresh material;/, no value; ND, not detected. The data are expressed as the mean ± SEM (n= 3). Different letters a, b, c, d within a row indicates statistically significant differences.

### 3.2 Microbial community of fresh oats and oat silage

**Figure 1:**
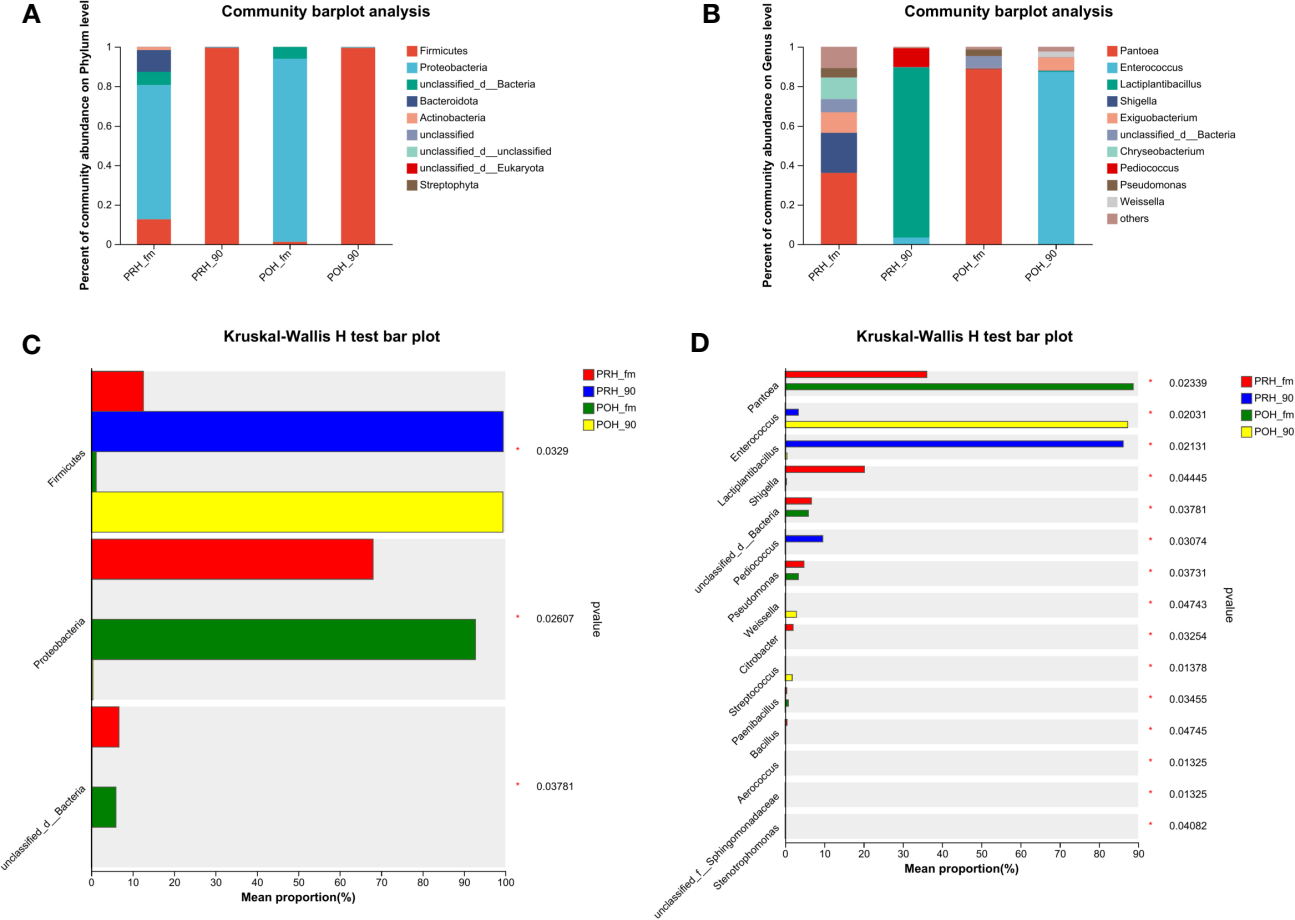
Relative abundance **(A, B)** and statistical comparison of relative abundance **(C, D)** of fresh and silage oat bacterial community at the phylum and genus levels. **(A)** Relative abundance at the phylum level. **(B)** Relative abundance at the genus level. **(C)** Statistical comparison of the relative abundance at the phylum level. **(D)** Statistical comparison of the relative abundance at the genus level. PRH-fm, the epiphytic microbiota of fresh oats at the pre-heading stage; PRH-90, the epiphytic microbiota of pre-heading oats after 90 days of fermentation; POH-fm, the epiphytic microbiota of fresh oats at the post-heading stage; POH-90, the epiphytic microbiota of post-heading oats after 90 days of fermentation.

**Figure 1** presents the microbiota composition at the phylum and genus levels in both fresh oats and oat silage. At the phylum level, Proteobacteria dominated the fresh oat samples harvested at both two stages, with abundances of (> 68%) and (> 92%). Firmicutes and unclassified d Bacteria were the next most dominant phylum found in the fresh oat samples. It was noteworthy that the abundances of Bacteroidota and Actinobacteria in the fresh oat samples were (> 11%) and (> 1%) at the pre-heading stage, while they were less abundant or not detected in fresh oat samples at the post-heading stage. After 90 days of fermentation, the most abundant phylum was Firmicutes, and almost all 16S sequences belonged to the phylum of Firmicutes in both stages (PRH-90 – 99.48% vs POH-90 – 99.43%).

Genus level compositions of the bacterial community in fresh oats and oat silage are described in **Figure 1B**. The most abundant genus in fresh oats was *Pantoea* at both stages, but there was a difference in its abundance (PRH-fm – 36.11% vs POH-fm – 88.74%). *Chryseobacterium* was detected in fresh oats at the pre-heading stage, while it was less abundant or not detected in fresh oats at the post-heading stage. Several other major genera detected in fresh oats were *Shigella*, *Exiguobacterium*, and *Pseudomonas*. Following a 90-day fermentation period, silages from raw materials harvested at different stages presented distinct microbial abundances. In pre-heading oat silage, the abundance of *Lactiplantibacillus* was the highest and dominant (> 86%), followed by *Pediococcus* (9.62%) and *Enterococcus* (3.37%), while in post-heading oat silage, the abundance of *Enterococcus* was the highest and dominant (> 87%), followed by *Exiguobacterium* (6.63%), *Weisella* (2.87%), and *Lactiplantibacillus* (0.46%).

As shown in **Figures 1C, D**, the relative abundance comparison bar chart of species was tested through the Kruskal-Wallis rank sum test. Following the ensiling process, there was a significant increase observed in the abundance of Firmicutes, whereas the abundance of Proteobacteria and unclassified d Bacteria exhibited a significant decrease compared to fresh oats (*p* < 0.05). Among the genera analyzed, *Pantoea*, *Enterococcus*, and *Lactiplantibacillus* exhibited the most significant variations in abundance at the genus level in **Figure 1D**. The abundance of *Pantoea* exhibited a significant increase following the ensiling process (*p* < 0.05). *Enterococcus*, *Lactiplantibacillus*, and *Pediococcus* significantly increased in pre-heading oat silage, and *Enterococcus*, *Weisella*, and *Streptococcus* significantly increased in post-heading oat silage (*p* < 0.05).

**Figure 2:**
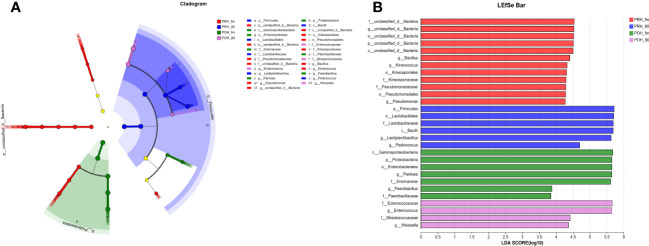
Evolutionary branch and LDA value distribution in fresh and silage oats harvested at different stages at the genus level OTU tables. **(A)** Evolutionary branch diagram of various species. The circles radiating from the inside to the outside represent classification levels ranging from phylum to genus. Different colored nodes indicate microbial taxa that are significantly enriched in the corresponding groups and that significantly affect the differences between groups. Light yellow nodes indicate microbial taxa that are not significantly different in any of the different groups or have no significant effect on the differences between groups. **(B)** LDA value distribution of different species (default score = 4). For LDA scores obtained by LDA analysis (linear regression analysis), the larger the LDA score, the greater the effect of species abundance on the differential effect. PRH-fm, the epiphytic microbiota of fresh oats at the pre-heading stage; PRH-90, the epiphytic microbiota of pre-heading oats after 90 days of fermentation; POH-fm, the epiphytic microbiota of fresh oats at the post-heading stage; POH-90, the epiphytic microbiota of post-heading oats after 90 days of fermentation.

To identify key biomarkers in oat silage, bacterial communities in oat silage were subjected to LEfSe analysis with a linear discriminant analysis threshold of 2.0. Among the bacterial community (**Figures 2A, B**), a total of 29 bacterial species, including notable genera such as *Bacillus*, *Lactiplantibacillus*, and *Enterococcus*, were identified as significantly different between the four groups. Three genera (*Bacillus*, *Kineococcus*, and *Pseudomonas*) in the PRH-fm group exhibited significant differences compared to the other three groups; two genera (*Pantoea* and *Paenibacillus*) in the POH-fm group exhibited significant differences compared to the other three groups; and two genera (*Lactiplantibacillus* and *Pediococcus*) in the PRE-90 group and (*Enterococcus* and *Weissella*) in the POH-90 group exhibited significant differences compared to the other three groups.

### 3.3 Relationships between nutritional components, fermentation products, and bacterial community

**Figure 3:**
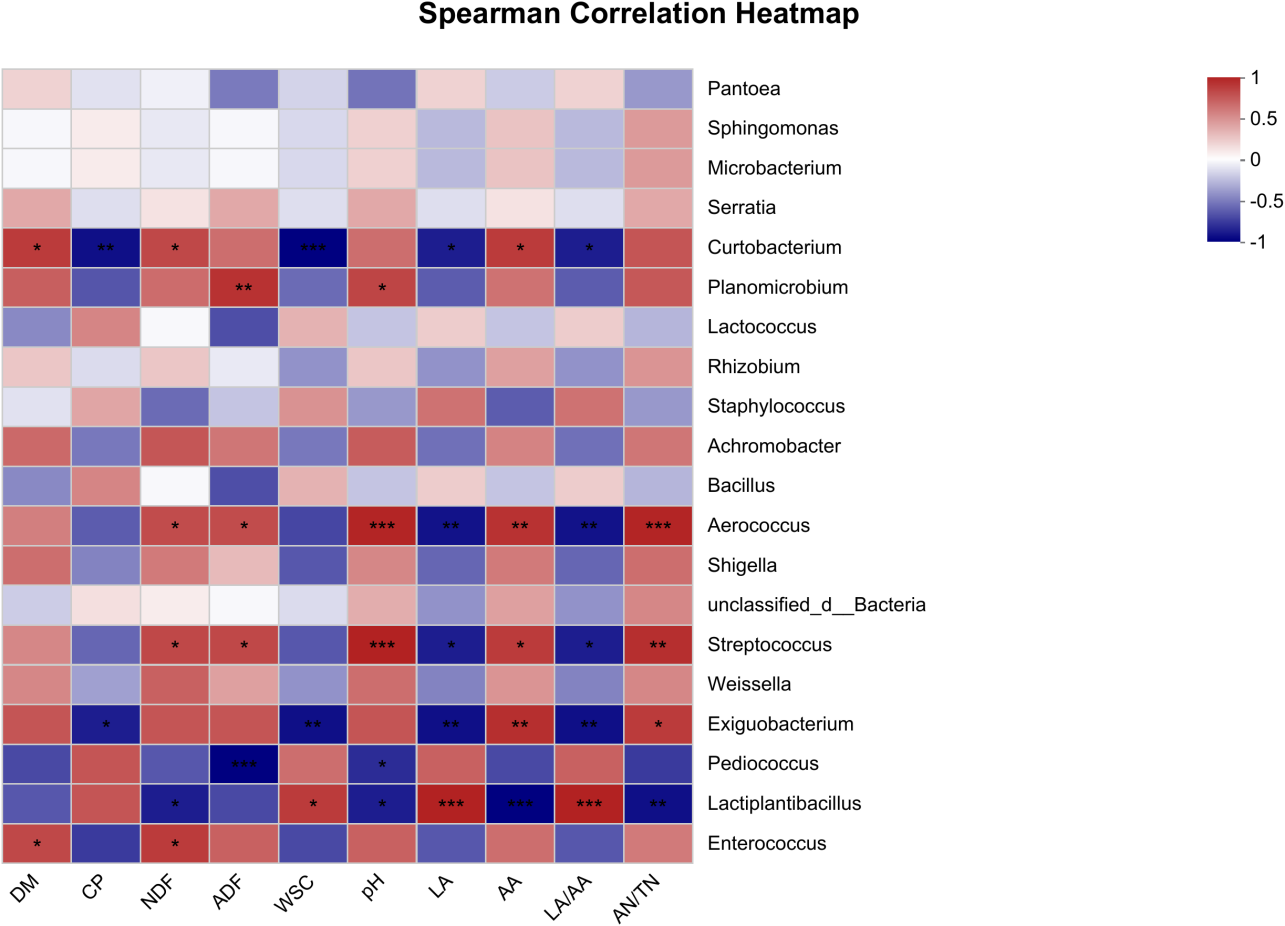
Spearman correlation heatmap of fermentative metabolites and bacterial community (top 20 genera) in oat silage fermented for 90 days. R-values are shown in different colors in the graph, with red indicating positive correlation (0 < *r* < 1) and blue indicating negative correlation (-1 < *R* < 0). The p-values are marked with * if 0.01 < *p* ≤ 0.05; **, 0.001 < *p* ≤ 0.01; ***, *p* ≤ 0.001.

The Spearman correlation heatmap which reveals the associations between fermentative metabolites and the microbial composition in oat silage is presented in **Figure 3**. The results show that the primary microorganisms engaged in the fermentation process were *Curtobacterium*, *Planomicrobium*, *Aerococcus*, *Streptococcus*, *Exiguobacterium*, *Pediococcus*, and *Lactiplantibacillus*. The CP content exhibited a significant negative correlation with *Curtobacterium* at *p* < 0.01 and *Exiguobacterium* at *p* < 0.05. The NDF content showed a positive correlation with *Curtobacterium*, *Aerococcus*, *Streptococcus*, and *Enterococcus*, while a negative correlation was observed with *Lactiplantibacillus* (*p* < 0.05). At a significance level of *p* < 0.001, the ADF content exhibited positive correlations with *Planomicrobium*, *Aerococcus*, and *Streptococcus*, while showing a negative correlation with *Pediococcus*. The WSC content was negatively correlated with *Curtobacterium* at *p* < 0.001 and *Exiguobacterium* at *p* < 0.01, while a positive correlation was observed with *Lactiplantibacillus* at *p* < 0.05. *Aerococcus* and *Streptococcus* showed positive associations with pH (*p* < 0.001), AA (*p* < 0.05), and AN/TN (*p* < 0.01), while exhibiting negative associations with LA (*p* < 0.05). *Exiguobacterium* was negatively associated with LA (*p* < 0.01), while a positive correlation was observed with AA at *p* < 0.01 and AN/TN at p < 0.05. *Lactiplantibacillus* exhibited a negative association with pH at *p* < 0.05, AA at *p* < 0.001, and AN/TN at p < 0.01, while demonstrating a positive association with LA and LA/AA (*p* < 0.001). It was noteworthy that *Planomicrobium* exhibited a positive correlation with pH (*p* < 0.05), AA (*p* > 0.05), and AN/TN (*p* > 0.05), but was negatively associated with LA and LA/AA (*p* > 0.05), while the opposite was true for *Pediococcus*.

### 3.4 Changes in volatile metabolites of oat silage at pre-heading and post-heading stages

In the current study, an extensive volatile metabolomics analysis was performed on oat silage samples collected at both pre-heading and post-heading stages, leading to the detection of a total of 662 volatile metabolites (**Supplementary Table 1**), including 30 acids, 50 alcohols, 44 aldehydes, 15 amines, 31 aromatics, 122 esters, 2 ethers, 3 halogenated hydrocarbons, 98 heterocyclic compounds, 60 hydrocarbons, 60 ketone, 7 nitrogen compounds, 14 phenols, 5 sulfur compounds, 119 terpenoids, and 2 others.

**Figure 4:**
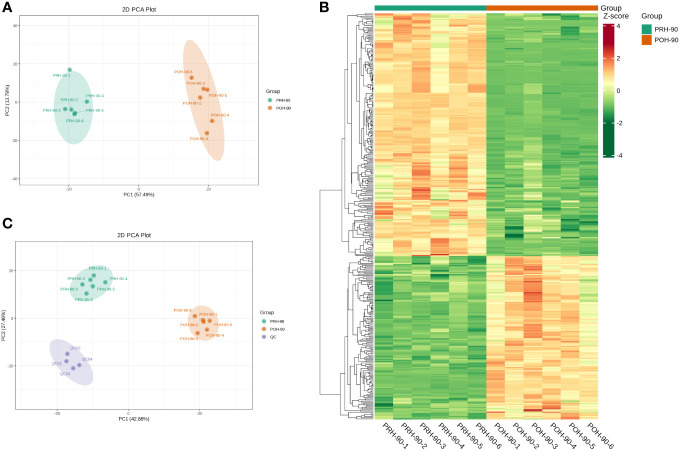
Multivariate statistical analysis of volatile metabolites from silage oats (PRH - 90 and POH - 90). **(A)** PCA scored plot of all silage oat samples. **(B)** Hierarchical cluster diagram of all silage oat samples. **(C)** PCA scored plot of all silage oat samples and the QC sample. PRH-90, volatile metabolites of pre-heading oats after 90 days of fermentation; POH-90, volatile metabolites of post-heading oats after 90 days of fermentation.

Unsupervised PCA was used to assess differences in volatile metabolites after fermentation of oats harvested at different stages. PCA effectively distinguished the PRH-90 and POH-90 samples from the QC samples, as depicted in **Figure 4C**. Additionally, the data points of the QC group exhibited a high concentration, suggesting a high level of repeatability in the sample collection process. Moreover, the oat silage samples were categorized into distinct regions based on the first principal component (PC1) and the second principal component (PC2), underscoring the impact of fermentation. PC1 (57.49%) and PC2 (13.78%) collectively accounted for 71.27% of the total variance (**Figure 4A**). Notably, the PRH-90 and POH-90 groups were significantly separated, indicating the substantial alteration of volatile metabolites in silage oats induced by fermentation.

Hierarchical cluster analysis (HCA) was conducted on the accumulation patterns of metabolites across different samples, resulting in the generation of an overall clustering map of the samples (**Figure 4B**). The heatmap clearly illustrated the distinct profiles of PRH-90 and POH-90, emphasizing the significant influence of microorganism activities during fermentation on the metabolites of oat silage.

**Figure 5:**
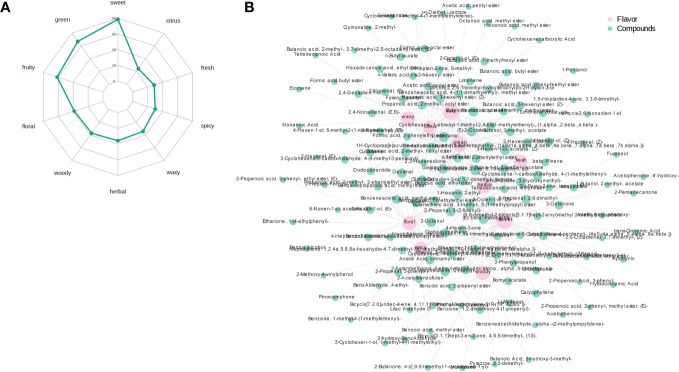
Radar chart of the sensory flavor profile of differential volatile metabolites and network chart of sensory flavor association with differential volatile metabolites. **(A)** Radar chart of the sensory flavor profile of differential volatile metabolites. The numbers corresponding to green dots are the number of differential metabolites annotated to that sensory flavor profile. **(B)** Network chart of sensory flavor association with differential volatile metabolites. The pink circle indicates the sensory flavor profile, the green circle indicates the differential metabolite, and the line between the two colored circles represents the differential metabolite annotated to that sensory flavor profile. When the number of annotated differential metabolites exceeds 50, the top 50 differential metabolites with the largest VIP values are displayed.

**Figure 5A** shows the radar chart of the top 10 sensory flavors selected for the highest number of annotations for the differential volatile metabolites and the annotated sensory flavor profiles obtained based on the screening criteria identified in the PRH-90 and POH-90 groups. The differential flavor metabolites were mainly enriched in sweet, green and fruity odors, with the most flavor substances indicating sweet odor reaching 56. **Figure 5B** shows the association network diagram of the top 10 sensory flavors with the corresponding differential flavor metabolites. Sweet odor was associated with Benzeneacetic acid, 2-methylpropyl ester, 2-Propenoic acid, 3-phenyl-, 2-Buten-1-ol, 3-methyl-, acetate, etc. The odor of green was associated with 2-Nonenal, (E)-, 2-Propenal, 3-(2-furanyl)-, Butanoic acid, 2-methyl-, hexyl ester, etc. The odor of fruity was associated with 2-Buten-1-ol, 3-methyl-, acetate, Butanoic acid, 2-methyl-, hexyl ester, Formic acid, octyl ester, etc. The above were only the top few differential volatile metabolites with the highest VIP value annotated to a sensory flavor profile.

**Figure 6:**
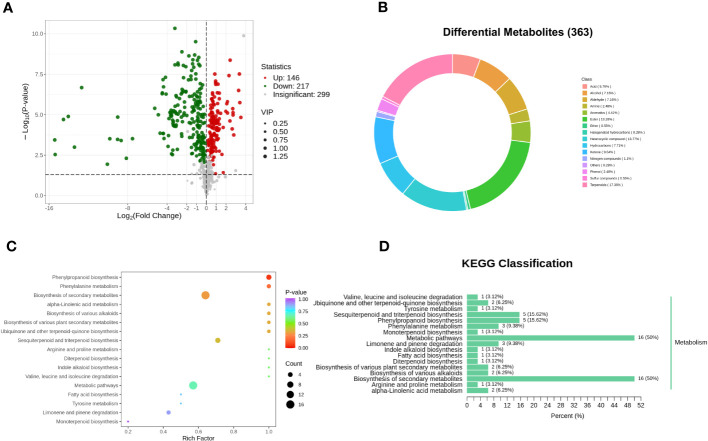
Differential volatile metabolites and metabolic pathways associated with differential volatile metabolites between PRH-90 and POH-90. **(A)** Volcano plot of differential volatile metabolites. **(B)** Grouping rings of differential volatile metabolites in terms of the number of classes. **(C)** Overview of KEGG pathway of the differential volatile metabolites. **(D)** Differential volatile metabolite KEGG classification plot.

In order to elucidate the disparities in volatile metabolites between oats at different fermentation stages, orthogonal partial least squares discrimination analysis (OPLS-DA) was conducted. This analysis aimed to identify the different volatile metabolites in PRH-90 and POH-90 samples using the criteria of VIP > 1 and a *P*-value < 0.05. A total of 363 differential volatile metabolites were screened between the PRH-90 and POH-90 samples (**Supplementary Table 2**). Among them, 146 volatile metabolites were up-regulated while 217 volatile metabolites were down-regulated, and these differentially expressed metabolites were visualized in a volcano plot to depict the distribution of volatile metabolites with significant changes (**Figure 6A**). **Figure 6B** showed the grouping ring of differential metabolite classes between PRH-90 and POH-90. The classes that account for a relatively high proportion of the differential volatile metabolites were ester (19.28%), terpenoids (17.36%), heterocyclic compound (13.77%), ketone (9.64%), hydrocarbons (7.71%), alcohol (7.16%), aldehyde (7.16%), and acid (5.79%).

### 3.5 Changes in metabolic pathways during fermentation

Pathway enrichment analysis of 363 differential volatile metabolites was performed using the KEGG database. A total of 32 differential volatile metabolites were identified, distributed in 17 metabolic pathways. Subsequently, we performed KEGG pathway enrichment analysis (**Figures 6C, D**) to determine the differences in metabolic pathways between the two groups of samples. The results of KEGG pathway enrichment analysis demonstrated three major metabolic pathways: phenylpropanoid biosynthesis, phenylalanine metabolism, and biosynthesis of secondary metabolites.

### 3.6 Relationships between bacterial community and volatile metabolites

**Figure 7:**
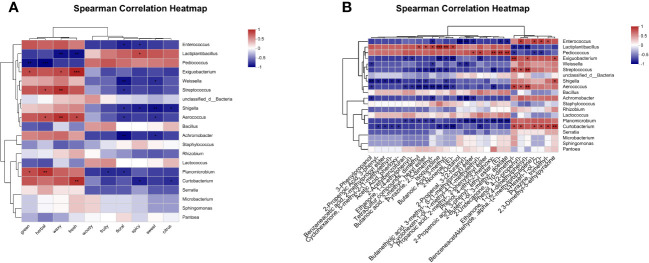
Correlation heatmap of bacterial community (top 20 genera) and differential metabolites after 90 days of fermentation. **(A)** Correlation between sweet, green, fruity, floral, woody, herbal, waxy, spicy, fresh, and citrus, as well as microorganisms. **(B)** Correlation between 28 flavor metabolites and microorganisms.

To clarify the effect of microbial activity on the flavor development of oats throughout fermentation, we screened the top 50 major differential volatile metabolites (VIP > 1 and *P*-value < 0.05) to obtain 28 flavor metabolites (**Supplementary Table 3**). Subsequently, Spearman correlation analysis was conducted to examine the correlation between the top 20 microorganisms in abundance and flavor metabolites (**Figure 7**). The results show that a total of 11 genera were related to the first 10 sensory flavor characteristics with higher annotations. In addition, 8 genera were associated with more than 9 flavor metabolites and 3 genera were associated with more than 4 flavor metabolites of less than 9 species.

**Figure 7A** illustrates the correlations between bacterial genus levels and the top 10 sensory flavor characteristics with extensive annotations. *Enterococcus* was positively correlated with green, herbal, waxy, and fresh odors (*p* > 0.05), while demonstrating a significant negative correlation with floral and spicy odors (*p* < 0.05). *Lactiplantibacillus* showed a positive correlation with spicy odor, but was negatively correlated with waxy and fresh odors (*p* < 0.05). *Pediococcus* showed a negative correlation with green (*p* < 0.01) and herbal (*p* < 0.001) odors. *Exiguobacterium* showed a positive correlation with green, waxy, and fresh odors (*p* < 0.05). *Streptococcus* showed a positive correlation with herbal and waxy odors, but was negatively correlated with floral odor (*p* < 0.05).

The correlations between bacterial genus levels and 28 flavor metabolites are shown in **Figure 7B**. *Enterococcus* showed a negative correlation with Pyrazine, 2,5-dimethyl-, 3-Octanol, 2-Propenal, 3-(2-furanyl)-, Butanethioic acid, 3-methyl-, S-(1-methylpropyl) ester, 2-Propenoic acid, 3-phenyl-, ethyl ester, (E)-, and 2-Buten-1-ol, 3-methyl-, acetate, but was positively correlated with Phenol, 4-(2-propenyl)-, Benzeneacetaldehyde,.alpha.-(2-methylpropylidene)-, 3-Hexen-1-ol, (Z)-, and Pyrazine, trimethyl- (*p* < 0.05). *Lactiplantibacillus* was positively correlated with TetraSulfur compounds, dimethyl, Butanoic acid, 2-methyl-, hexyl ester, Pyrazine, 2,5-dimethyl-, Isoquinoline, Butanoic Acid, 3-methyl-, and 2-Nonenal, (E)-, but negatively correlated with 2-Undecanone, 6,10-dimethyl-, Phenol, 4-(2-propenyl)-, and Ethanone, 1-(2,4-dimethylphenyl)- (*p* < 0.05). *Pediococcus* showed a positive correlation with Butanethioic acid, 3-methyl-, S-(1-methylpropyl) ester, 3-Cyclohexen-1-ol, 1-methyl-4-(1-methylethyl)-, Phenol, 4-ethyl-2-methoxy-, 2-Propenoic acid, 3-phenyl-, ethyl ester, (E)-, and 2-Buten-1-ol, 3-methyl-, acetate, but was negatively correlated with 2-Undecanone, 6,10-dimethyl-, Benzeneacetaldehyde,.alpha.-(2-methylpropylidene)-, and 3-Hexen-1-ol, (Z)- (*p* < 0.05). *Exiguobacterium* was negatively correlated with 2-Acetylbenzofuran, Ethanone, 1-(4-ethylphenyl)-, Isoquinoline, Butanoic acid, 3-methyl-, 2-Nonenal, (E)-, and 3-Cyclohexen-1-ol, 1-methyl-4-(1-methylethyl)-, but negatively correlated with 2-Undecanone, 6,10-dimethyl-, Ethanone, 1-(2,4-dimethylphenyl)-, and 2,3-Dimethyl-5-ethylpyrazine (*p* < 0.05). *Streptococcus* was negatively correlated with Tetrasulfur compounds, dimethyl, Butanoic acid, 2-methyl-, hexyl ester, Pyrazine, 2,5-dimethyl-, Isoquinoline, Butanoic acid, 3-methyl-, and Phenol, 4-ethyl-2-methoxy-, but positively correlated with 2-Undecanone, 6,10-dimethyl-, Phenol, 4-(2-propenyl)-, and Ethanone, 1-(2,4-dimethylphenyl)- (*p* < 0.05).

## 4 Discussion

Nutritional components, fermentation products, and microbial population differed in oats harvested at pre-heading and post-heading stages. As the fertility of oats increased, water content decreased and dry matter accumulation increased, which led to a gradual decrease in nutritional components, as indicated by the decrease in CP, NDF, and ADF contents. The increase of WSC content in post-heading oats may be due to the gradual accumulation of starch in the seeds as the plant matures (David et al., 2010). However, the WSC content in fresh oats at both stages was lower than the value suggested by Amer et al. (2012), and may not provide sufficient fermentation substrate for lactic acid bacteria, leading to slow growth and inability of lactic acid bacteria to dominate fermentation process. The microbial community attached to the raw material plays a crucial role in determining the quality of silage (Lin et al., 1992). In this study, a low number of lactic acid bacteria were found to be attached to fresh oats, but the count of aerobic bacteria was high, and a large number of coliform bacteria were attached to fresh oats, which are adverse for silage fermentation. Epiphytic populations of yeasts and molds were within the range of values typically reported before ensiling (Pahlow et al., 2003).

After ensiling, CP, NDF, ADF and WSC contents showed a decrease. The decrease in CP content is due to the hydrolysis of true proteins into peptide nitrogen, free amino acid nitrogen, NH3-N and non-protein nitrogen such as amines through the synergistic action of plant proteases and microbial enzymes (Bommert and Whipple, 2018). The decrease in NDF, ADF and WSC content was due to the decomposition of some cellulose and hemicellulose during silage fermentation, which were consumed as substrates together with WSC by microorganisms (Dean et al., 2005). The pH value serves as a crucial indicator of silage quality, providing insights into the preservation status and the degree of decomposition caused by undesirable microorganisms (Ren et al., 2022). In the present study, the pH level of pre-heading oat silage was significantly lower than that of post-heading oat silage (4.73 vs 5.57), which may be due to the high content of unfermentable components such as NDF and ADF in oats at the post-heading stage, which in turn affects the fermentation of oats by microorganisms. Fresh oats at the post-heading stage contained more miscellaneous bacteria, which led to the inability of lactic acid bacteria to dominate in the fermentation and high pH due to poor microbial fermentation. Correspondingly, compared to oats ensiled at the pre-heading stage, oat silage at the post-heading stage contained lower LA (30.43 g/kg DM) and higher AN/TN (9.06). The ratio of AN/TN in silage indicated the extent of protein and amino acid decomposition, and lower values of this ratio indicate less protein decomposition and better silage quality (Yuan et al., 2012). The differences in LA, AA, and AN/TN among the oat silages of both two stages in this study may be related to the type and number of microorganisms attached to the oats at the time of harvesting.

Further microbial sequencing analysis confirmed the experimental results. Throughout the fermentation process of 90 days, there was a shift in the dominant bacterial phylum from Proteobacteria and Bacteroidota to Firmicutes, which is a common occurrence in silage (Siran et al., 2020; Bai et al., 2023). This shift could be attributed to the acidic and anaerobic conditions during the ensiling process, which created a more favorable environment for the proliferation of Firmicutes (Keshri et al., 2018). The bacterial community composition at the genus level was significantly different between the two treatment groups, with *Lactiplantibacillus* dominating the PRH-90 group and *Enterococcus* dominating the POH-90 group. The reason for this finding may be due to the different raw materials and the different microorganisms attached to the raw materials. The PRH-90 group exhibited successful fermentation due to the significant role of *Lactiplantibacillus* in inhibiting the growth of undesirable microorganisms, reducing the ammoniacal nitrogen content, and enhancing the quality of the silage (Du et al., 2022). The POH-90 group contained more undesirable microorganisms on the raw materials and the pH dropped slowly, failing to provide the acidic environment needed for a well-fermented silage. Wang et al. (2019) reported that the naturally fermented treatment group exhibited a higher abundance of *Enterococcus*, which resulted in increased AA and NH_3_-N content, while negatively impacting silage quality. Similarly, in this study, the POH-90 group had poorer silage quality due to the dominance of *Enterococcus* in the fermentation, resulting in lower LA content, and higher pH, AA content and NH_3_-N/TN compared to the PRH-90 group.

Because the main process of silage fermentation is due to the activities of microorganisms, investigating the relationship between nutritional components, fermentation products, and microorganisms is vital for comprehending their role in the fermentation process. In the present study, a notable association was identified between eight genera and nutritional components, as well as fermentation products. The highest abundance of *Lactiplantibacillus* was found in the PRH-90 group and was positively correlated with WSC and LA, and negatively correlated with pH, AA and AN/TN. This showed that *Lactiplantibacillus* could better preserve WSC, increase LA, and lower pH and AN/TN, thus achieving good nutrient preservation and improving silage quality (Dong et al., 2022). Lower pH and AN/TN are usually better, as low pH could inhibit harmful microorganisms, and AN/TN could reflect the degree of protein degradation during silage process. Well-fermented silage feed generally has low pH and AN/TN (Filya, 2003). *Pediococcus* was the second most abundant genera detected in the PRH-90 group and also showed a negative correlation with pH, probably due to its synergistic effect with *Lactiplantibacillus*. Liu L. et al. (2022) reported that the dominance of *Lactiplantibacillus* on day 31 of the fermentation process was accompanied by a significant rise in the abundance of *Pediococcus*. In the POH-90 group, *Enterococcus* was the most abundant genera, but there was no significant association with indicators of silage quality. Cai (1999) inoculated samples with *Enterococcus* as an additive and found that *Enterococcus* did not improve the fermentation quality of the silage. The detection of *Exiguobacterium*, *Streptococcus*, and *Aerococcus* in the POH-90 group samples showed a positive correlation with the level of AN/TN, while *Streptococcus* and *Aerococcus* also showed a positive correlation with pH, which may explain the higher levels of pH and AN/TN in the POH-90 group compared to the PRH-90 group.

The odor of silage could reflect the quality of silage more directly, and volatile metabolomics has been widely used to detect flavor chemistry (Qualley and Dudareva, 2009). Ester and terpenoids were found to be the main volatiles of silage in this study, which is consistent with other studies (Di Cagno et al., 2017; Weiss, 2017). Analysis of differential volatiles in the PRH-90 and POH-90 groups detected a total of 363 differential volatiles, indicating that the flavors of the PRH-90 and POH-90 groups were significantly different, which is consistent with the PCA results, where the two groups were clearly separated, implying that the two groups were significantly different. Compared with the PRH-90 group, the content of many esters in the POH-90 group decreased significantly, while flavor substances indicating sweetness and fruitiness, such as Benzoic acid, ethyl ester, 1-Butanol, 3-methyl-, acetate, Pentanoic acid, 4-methyl-, methyl ester, Formic acid, octyl ester, and Benzeneacetic acid, 2-methylpropyl ester, which could make the fermentation more intense in terms of fruity and floral odors, were the main contributing components to the aroma of the silage (Shukui et al., 2007; Fernando et al., 2008; Sympoura et al., 2009; José, 2014). Terpenoids have been previously associated with citrus and pine odors, and an increase in the content of these compounds may enhance the fragrance (Beaulieu et al., 2015). The content of terpenoids with citrus and pine odors (2,6-Octadienal, 3,7-dimethyl-, (Z)-, Limonene oxide, cis-, 2,7-Octadien-4-ol, 2-methyl-6-methylene-, (S)-, etc.) in the POH-90 group also decreased. In addition, there was a decrease in flavor substances among acids, alcohols, ketones, aldehydes and heterocyclic compounds contributing components to the aroma. KEGG pathway enrichment analysis demonstrated metabolic pathways that may be associated with differential metabolite synthesis. The most significant metabolic pathways included phenylpropanoid biosynthesis, phenylalanine metabolism, and biosynthesis of secondary metabolites. The phenylpropanoid biosynthesis pathway is related to phenolic acid substances, and phenylalanine metabolism is an important metabolic pathway in the winemaking process and is connected to the generation of aromatic compounds (Fairbairn et al., 2017; Wang et al., 2022). In this experiment, the content of substances such as Phenol, 2-methoxy-4-(1-propenyl)-, trans-Cinnamic acid, and 2-Propenoic acid, 3-phenyl-, which represent sweet and floral odors, decreased in the POH-90 group. These substances are usually influenced by the phenylpropanoid biosynthesis and phenylalanine metabolism pathway. The biosynthesis of secondary metabolites pathway was related to the synthesis of terpenoids, and the large number of terpenoids detected in this experiment may have been influenced by the biosynthesis pathway of secondary metabolites (De et al., 2011). These findings could potentially elucidate the variation in odors observed between the PRH-90 and POH-90 groups following fermentation.

The formation of metabolites in silage is intricately linked to the presence and activities of microorganisms, and this experiment further analyzed the relationship between the dominant microorganisms (top 20 genera) and odors as well as the main differential flavor substances. The dominant genera (*Lactiplantibacillus* and *Pediococcus*) in the well-fermented PRH-90 group were positively correlated with odors that mostly indicated good flavor such as sweet, fruity, floral, and citrus, while the dominant genera (*Enterococcus*, *Exiguobacterium*, *Weisella*, and *Streptococcus*) in the poorly fermented POH-90 group were negatively correlated with odors that indicated poor flavor such as green, herbal, waxy and fresh, indicating that microorganisms play a pivotal role in shaping the final flavor of silage through their activities during the fermentation process. Butanoic acid, 2-methyl-, hexyl ester, 2-Nonenal, (E)- and 2-Propenoic acid, 3-phenyl-, ethyl ester, (E)- have been reported as aroma substances with a large impact on flavor (Palma-Harris et al., 2001; Young et al., 2004; Pu et al., 2021), and in this experiment they may have been influenced by the fermentation of *Lactiplantibacillus* and *Pediococcus*, and had an important effect on the formation of fruit odor in silage. 2-Propenoic acid, 3-phenyl-, ethyl ester, (E)- and 2-Buten-1-ol, 3-methyl-, acetate have been reported as aroma substances that, contribute significantly to the fermentation consequent aroma, and their synthesis may have been promoted by the fermentation of *Pediococcus* in this experiment, while their synthesis may have been inhibited by the fermentation of *Enterococcus*. Previous studies have shown that *Lactiplantibacillus* and *Pediococcus* contribute to the synthesis of aroma substances during fermentation, suppress undesirable odors and improve the flavor of fermented products, while the fermentation process dominated by *Enterococcus* could produce undesirable odors (Miller et al., 2018; Huipeng et al., 2020; Zhen et al., 2020). However, our results could not fully elucidate the role of microorganisms in the metabolism of flavor substances, and the metabolic mechanisms of flavor substances and the role of microorganisms in them need to be further explored and elucidated.

## 5 Conclusion

To investigate the changes in silage quality and flavor of oats harvested at pre-heading and post-heading stages, microbial diversity and volatile metabolites were analyzed throughout the oat fermentation process. The results indicated that PRH-90 group was dominated by *Lactiplantibacillus* with lower pH and AN/TN and better fermentation quality compared to POH-90 group, while POH-90 group was dominated by *Enterococcus* with higher pH and AN/TN and poorer fermentation quality compared to PRH-90 group. Esters and terpenoids were compounds that significantly contributed to flavor during oat fermentation. The differences in flavor between the two groups of oats after ensiling were mainly concentrated in sweet, green, and fruity odors. Changes in the relative abundance of major flavor metabolites were highly correlated with microorganisms. *Lactiplantibacillus* and *Enterococcus*, as the predominant genera in the oat fermentation process, are considered to contribute significantly to the flavor quality of silage oats. The results of this study provide insights into the relationship between microorganisms and flavor development during oat fermentation, and help to further understand the regulatory mechanisms of silage flavor formation.

## Data availability statement

The datasets presented in this study can be found in online repositories. The names of the repository/repositories and accession number(s) can be found below: SRA data: PRJNA1005624 (including 12 SRA (sequence read archive) accession numbers: SRX21369993- SRX21370004).

## Author contributions

XD: Conceptualization, Methodology, Investigation, Data curation, Formal analysis, Validation, Visualization, Writing – original draft. YJ: Funding acquisition, Methodology, Project administration, Supervision, Writing – review & editing. GG: Methodology, Project administration, Supervision, Writing – review & editing. ZW: Methodology, Writing – review & editing. ML: Methodology, Writing – review & editing, Conceptualization, Resources, Supervision. JB: Methodology, Writing – review & editing. MZ: Methodology, Writing – review & editing. QS: Methodology, Writing – review & editing. YL: Methodology, Writing – review & editing. WZ: Methodology, Writing – review & editing.

## Supplementary material

Supplementary Table 1 Volatile metabolites detected by GC-MS.

Supplementary Table 2 Differential volatile metabolites detected by GC-MS.

Supplementary Table 3 The flavor substances in the top 50 differential metabolites identified by VIP (VIP > 1.0) and P-value (P-value < 0.05).

Supplementary Table 4 Flavor substances contained in the top 10 highest number of sensory flavor profiles annotated to differential metabolites identified by the comparison group based on screening criteria.
